# Age-Related Trajectories of General Fluid Cognition and Functional Decline in the Health and Retirement Study: A Bivariate Latent Growth Analysis

**DOI:** 10.3390/jintelligence11040065

**Published:** 2023-03-29

**Authors:** Elizabeth P. Handing, Yuqin Jiao, Stephen Aichele

**Affiliations:** 1 Department of Human Development and Family Studies, Fort Collins, CO 80523, USA; 2 Colorado School of Public Health, Fort Collins, CO 80523, USA

**Keywords:** cognitive aging, functional limitations, latent trajectory, bivariate, longitudinal, middle age

## Abstract

There have been few studies on associations between age-related declines in fluid cognition and functional ability in population-representative samples of middle-aged and older adults. We used a two-stage process (longitudinal factor analysis followed by structural growth modeling) to estimate bivariate trajectories of age-related changes in general fluid cognition (numeracy, category fluency, executive functioning, and recall memory) and functional limitation (difficulties in daily activities, instrumental activities, and mobility). Data came from the Health and Retirement Study (Waves 2010–2016; N = 14,489; ages 50–85 years). Cognitive ability declined on average by −0.05 SD between ages 50–70 years, then −0.28 SD from 70–85 years. Functional limitation increased on average by +0.22 SD between ages 50–70 years, then +0.68 SD from 70–85 years. Significant individual variation in cognitive and functional changes was observed across age windows. Importantly, cognitive decline in middle age (pre-age 70 years) was strongly correlated with increasing functional limitation (*r* = −.49, *p* < .001). After middle age, cognition declined independently of change in functional limitation. To our knowledge, this is the first study to estimate age-related changes in fluid cognitive measures introduced in the HRS between 2010–2016.

## 1. Introduction

Successful aging and autonomy in later adulthood depend critically on the preservation of cognitive abilities and physical functioning (Fiocco and Yaffe 2010). There is robust evidence both from cross-sectional and longitudinal studies showing that cognitive performance and physical functioning decline with increasing age and that these declines are interrelated (Demnitz et al. 2016; Montero-Odasso et al. 2019; Peel et al. 2019). For example, in meta-analyses by Demnitz et al. (2016) (*n* = 26 studies) and Peel et al. (2019) (*n* = 36 studies), the majority of studies reported a significant association between slower gait speed and worse cognitive function in older adults. The number, consistency, and quality of studies provide strong evidence for a significant association. There is also evidence that such declines, especially in cognitive abilities, accelerate in later-adulthood and/or in proximity to death (Gerstorf et al. 2013). In other words, changes in cognitive and functional abilities, and associations between changes in these abilities, likely vary across the adult lifespan, e.g., during middle age vs. late adulthood. This latter possibility has been underexplored insofar as population-representative studies with samples of middle-aged adults often lack cognitive measures sensitive to normative age-related change (Salthouse 2009), whereas studies that do include such measures are often limited to older (post-middle-age) adults. Here, we sought to address this gap by modeling bivariate trajectories of changes in general fluid cognition and physical functioning in a broadly representative sample of adults from the Health and Retirement Study (HRS).

The HRS is one of multiple country-level efforts to track changes in socioeconomic and health-related measures in population-representative samples of middle-aged and older adults, repeatedly assessed at 2-year intervals. A compelling feature of these studies is the inclusion of harmonized measures of physical and mental health (Gateway to Global Aging Data; accessed on 15 October 2022, https://g2aging.org/, National Institute on Aging n.d.) which opens possibilities for pooling and comparing information across countries. However, cognitive variables in most of these studies to date have been limited to relatively simple measures that could be easily administered by phone interview. Recognizing this limitation, several psychometricians convened in 2003 (Fisher et al. 2014) to introduce additional cognitive measures in the HRS to better assess fluid abilities (i.e., abstract reasoning, frontal/executive functioning), which are more sensitive to the effects of normative aging yet also comparatively predictive of critical later-life health outcomes (e.g., mortality risk, depression risk) (Aichele et al. 2015, 2019). Data from these measures are available in the 2010, 2012, and 2016 HRS waves but have seldom been analyzed.

More generally, studies examining relations between cognitive and physical functioning in older adults have mostly leveraged coarse and/or singular measures of cognitive “status” (e.g., the Mini Mental Status Exam, or MMSE) and functional ability (e.g., gait speed or grip strength) (Collyer et al. 2022; Deary et al. 2011; Gonzales et al. 2020; Jayakody et al. 2021; Montero-Odasso et al. 2020, 2018; Robitaille et al. 2018; Tian et al. 2020; Zammit et al. 2019). Longitudinal cognition-functional health studies have typically focused on samples of older adults (aged over 65 years) (Ritchie et al. 2016). In a systematic review, Clouston et al. (2013) identified 15 longitudinal studies on fluid ability and physical functioning. Out of these, eight included samples with an age range spanning middle-adulthood, nine included samples with approximately proportional representation of women and men (i.e., ratios ranging from 40/60 to 60/40), and eight had sample sizes of N > 1000. Only three of these studies met all of these criteria. Results across these latter studies linked fluid ability at baseline with grip strength (Aichberger et al. 2010), lung function (Richards et al. 2005), and midlife declines in fluid ability with chair rising (Kuh et al. 2009). Only one of the 15 identified longitudinal cognition-physical functioning studies included more than a single measure of fluid ability (Tabbarah et al. 2002), and none assessed difficulties in activities of daily living as a facet of physical functioning.

Moreover, nearly all longitudinal cognition-functional health studies have modeled change as a function of time-in-study rather than as a function of chronological age. One exception was a study by van der Willik et al. (2021), which showed that cognition (including measures of word fluency, letter-digit substitution, Stroop test, and immediate and delayed word recall) and motor function (gait, finger dexterity, and fine motor skills) declined linearly between the ages of 45 and 65 years, followed by a steeper decline after the age of 65–70 (n = 9514 participants from the Rotterdam Study). Bivariate cognitive-functional change associations were not examined.

In summary, there is at present a dearth of longitudinal bivariate studies using sensitive, multi-domain measures of cognitive and physical functioning that accommodate meaningful “life course” interpretations of change associations from middle age into later adulthood. To address these limitations, we used longitudinal structural equation models to examine associations between age-related changes in general fluid cognition (as indicated by measures of numeracy, category fluency, executive function, and verbal recall memory) and in functional limitation (as indicated by difficulties in daily living activities, instrumental activities, and mobility) in a sample of middle-aged and older adults (N = 14,489). We compared linear and non-linear spline models of change (the latter with knot points at ages 60, 64, 70, and 74 years, respectively). In light of prior evidence, we hypothesized that the non-linear spline models, which accommodate different bivariate change associations across different age windows (e.g., in middle age vs. later adulthood), would best fit the data. We further expected that age-related increases in functional limitation would be linked with cognitive decline and that this association would be more pronounced in later (vs. earlier) adulthood.

## 2. Materials and Methods

### 2.1. Participants

Participants came from the Health and Retirement Study (HRS), a biannual and nationally representative survey of middle-aged and older adults (aged 50 years and older) from the United States. The HRS is sponsored by the National Institute on Aging and is conducted by the University of Michigan. For the purposes of this study, longitudinal data were drawn from the 2010, 2012, and 2016 waves of the HRS. These waves were selected because improved measures of adults’ fluid cognitive abilities were introduced in the HRS in 2010 and subsequently assessed at the 2012 and 2016 waves (Fisher et al. 2014). Note that some of the participants for the current studies entered the HRS prior to 2010: Time-invariant information for these individuals (e.g., biological sex, years of education) may come from earlier waves. Sample descriptive statistics are shown in Table 1.

The general inclusion criteria for the current study were that participants: (a) were without a prior medical diagnosis of Alzheimer’s Disease or dementia, (b) showed no evidence of having had a major depressive episode within the year prior to and including the time of study entry, and (c) were of age 50–85 years at first assessment (observations were sparse outside of that age range).

### 2.2. Measures

Measures for the analyses included four cognitive assessments and three functional limitation assessments (per wave).

#### 2.2.1. Cognitive Assessments

Several new cognitive tests were added to the HRS in 2010 to better assess fluid cognitive functioning (Fisher et al. 2014). In the present study, measures of numeracy, category fluency, executive functioning, and recall memory tests were selected because they have been shown to be sensitive to age-related effects, and have also been linked to elevated risks for cognitive impairment and dementia, depression, and all-risk mortality (Aichele et al. 2018; Johnson et al. 2007; McDonough et al. 2016). Here, we further operationalized general fluid cognition as a global latent measure (factor) indicated by performance on these cognitive tests (McArdle et al. 2007).

Numeracy. The HRS first introduced this number series task in 2010. It is adapted from the Woodcock-Johnson (WJ-R) tests of cognitive ability (Fisher et al. 2014). This task utilizes quantitative reasoning and numerical knowledge (i.e., non-verbal/fluid problem-solving) to correctly identify the missing number in a number series that is read aloud. All participants started with the same first 3-item block, which included an easy item, a moderately difficult item, and a more difficult item. Participants were then asked to answer one of the 4 remaining sets of 3-item blocks based on the number of items answered correctly in the first block. In total, each participant was expected to answer 6 items. The total score for the number series task from each wave was provided by the HRS as a standardized W-score adjusted for the difficulty level of the items asked. The scoring details (e.g., W-scale and scoring tables) for the number series task can be found in Fisher et al. (2014). The average within correlation of scores from 2010–2016 was 0.60. Documentation from the full HRS sample shows a within correlation of 0.59 (Fisher et al. 2014).

Category fluency. The retrieval fluency task was also introduced in 2010 (Fisher et al. 2014). Each participant was asked to recall as many animal names as possible within 60 s. The total score was calculated by subtracting the number of incorrect responses from the total number of animals named. Category fluency spans several cognitive dimensions, including processing speed and retrieval, and has consistently been linked to mild cognitive impairment (Yeung et al. 2016). The average within correlation of scores from 2010–2016 was 0.56. Documentation from the full HRS sample shows a within correlation of 0.57 (Fisher et al. 2014).

Executive function. The Serial 7s test has been widely used in previous research and is a relatively simple assessment of concentration and working memory (Folstein et al. 1975). Each participant was asked to subtract 7 from 100 in a serial fashion for a total of five trials. The participant was expected to remember the value from each subtraction and instructed to continue the subtraction procedure until they finished 5 trials. The number of correct subtractions was recorded as the final score for this task (0–5). (McCammon et al. 2022). The average within correlation of scores from 2010–2016 was 0.42. Documentation from the full HRS sample shows a within correlation of 0.66 (Fisher et al. 2014).

Recall memory. Recall memory was measured by immediate and delayed word recall tests. The interviewer read a list of 10 words to the participant one word at a time. The immediate recall score was calculated as the total number of correct words immediately recalled after the participant was presented with all the words. The delayed recall score was calculated as the total number of words correctly recalled approximately 5 min after the immediate word recall test. The total word recall score was created by combining immediate recall (score: 0–10) and delayed recall (score: 0–10) scores with a range from 0 to 20 for each of the three waves (McCammon et al. 2022). The average within correlation of scores from 2010–2016 was 0.42 and the results in the full HRS sample was 0.49 (Fisher et al. 2014).

#### 2.2.2. Functional Limitation

Functional limitation was evaluated using three multi-item self-report measures: (a) mobility, strength, and fine motor skills; (b) ability to perform activities of daily living (ADLs); (c) ability to perform instrumental activities of daily living (IADLs). The participants responded to items on each measure with yes/no, can’t do, or don’t do. The score for each question was recorded as yes = 1 and no = 0 (including no, can’t do, and don’t do); all other options were coded as missing (including don’t know, not ascertained, refused, and missing). The test-retest reliability is very good for ADLs and IADLs (ADL *r* = .59, IADL *r* = .93) (Edwards 1990). Additionally, self-reported measures of ADLs and IADLs have been shown to correlate with objective physical measures, with correlations ranging from 0.30–0.54 (Bravell et al. 2011).

Mobility, strength, and fine motor skills. Participants were asked if they had any difficulty with mobility (e.g., walking one block, walking several blocks, jogging 1 mile, getting up from a chair, climbing several flights of stairs, sitting for 2 h, stooping, reaching arms above shoulders), strength (e.g., lifting and carrying 10 pounds, pull/push a large object), and fine motor skills (e.g., picking a dime up) (Fonda and Herzog 2004). Items were summed and a higher score means more limitations, score range 0–11.

Activities of daily living (ADLs). Participants were asked to indicate if they had any difficulties with ADLs (needed for basic unassisted living), including difficulties in walking, dressing, bathing, eating, getting in/out of bed, and using the toilet. A total score was calculated by adding the number of items endorsed, a higher score means more limitations, score range 0–6 (Fonda and Herzog 2004).

Instrumental activities of daily living (IADLs). Participants were asked to indicate if they had any difficulties performing more complex daily tasks, including preparing meals, grocery shopping, managing money, taking medications, making telephone calls, (Fonda and Herzog 2004). Items were summed and higher values mean more limitations, range 0–5.

### 2.3. Analyses

We used a two-stage procedure to estimate stable individual differences and longitudinal changes in cognition and functional limitation. For these analyses, we used R statistical software v4.1.2. (R Core Team 2021). Structural equation models were estimated using the Lavaan package (Rosseel 2012) with full information maximum likelihood (FIML) to handle missingness and with the Huber-White robust estimator (Huber 1967). Absolute model fit was evaluated based on the Tucker-Lewis index (TLI) and the root mean square error of approximation (RMSEA) (Hu and Bentler 1999; Steiger 1990). Relative model fit was evaluated based on changes in chi-square (deviance) per changes in degrees of freedom and based on Akaike’s information criterion (AIC). Significance of parameter estimates was evaluated using an alpha criterion of α = 0.005 due to the large sample size.

#### 2.3.1. Longitudinal Factor Analysis

In the first stage, we used longitudinal factor analysis (LFA) to summarize individuals’ cognitive ability and functional limitation as latent scores (also known as factors) at each of the observed HRS measurement waves (2010, 2012, and 2016). Cognitive ability was indicated by performance on four tasks: numeracy, category fluency, executive function, and recall memory. Functional limitation was indicated by three measures: difficulty performing ADLs, difficulty performing IADLs, and difficulties with mobility, strength, or fine motor actions. Firstly, we ran independent factor analyses for cognitive and functional limitation variables, scaled in their original metrics, and imposed strong factorial invariance (e.g., constraining item-factor loadings and manifest variable intercepts across waves) to ensure consistency of item-factor representations/scaling (Widaman et al. 2010). We then standardized the cognitive and functional limitation variables using the corresponding mean and SD at the 2010 measurement wave (for consistent scaling to support model convergence) and re-estimated the cognitive and functional limitation factors concurrently in a bivariate longitudinal factor model, again imposing strong factorial invariance. Individuals’ factor scores at each wave were then extracted from the model results using Bartlett’s method (Devlieger et al. 2016). Full details of the LFA, including histograms and correlation tables for the observed variables, are provided in the Supplemental Materials (SM).

#### 2.3.2. Latent Trajectory Models

In the second stage, we estimated a series of univariate trajectory models for cognitive ability and functional limitation. The change was operationalized based on chronological age in decades (rather than measurement wave) as the time metric. As HRS waves took place at 2-year intervals, we used a “time window” approach (Grimm et al. 2017; McArdle et al. 2002) wherein we placed individuals’ factor scores (estimated in the first analysis stage) into corresponding 2-year “age bins”, beginning at age 50 (e.g., age bin 50/51) and extending to age 85 (e.g., age bin 84/85). We did not model the trajectories beyond age 85 years due to data sparseness at later ages (<5% observations per age bin).

We fit five models to each process: (a) an intercept-only model; (b) intercept + linear slope; (c–f) intercept + spline, with two slopes estimated pre- and post-knot points at age bins of (c) 60/61 years, (d) 64/65 years, (e) 70/71 years, and (f) 74/75 years, respectively. A knot point of 64/65 years was first chosen to correspond with the traditionally ascribed beginning of older age (ending of middle age) and also for consistency with average participant age at the initial wave (year 2010). Additional knot points at +/−5-year intervals on either side were then selected to test for earlier vs. later declines in cognition and functional ability. The final knot at 74/75 years was also tested given that accelerating cognitive declines often manifest in this age range.

**Figure 1:**
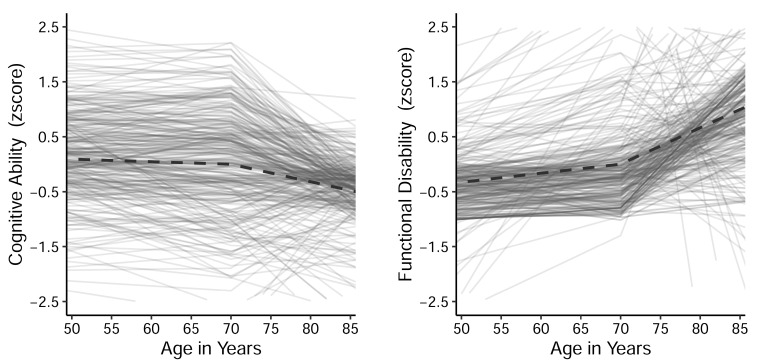
Group average trajectories (dark, dashed lines) estimated from univariate models are shown, accompanied by estimated individual trajectories for a sub-sample of study participants (*n* = 300; light-colored solid lines. Trajectories are shown scaled based on the means and SDs at age 70 years.

We estimated a bivariate latent trajectory model based on the best-fitting longitudinal parameterizations from the univariate models (BLTM; Figure 1). To further assist interpretation, the factor scores for the BLTM were re-scaled (standardized) based on means and SDs at age 70 years (e.g., age bin 70/71)—note that this simply allowed for interpretation of changes in terms of standardized units and had no effect on model fit statistics.

## 3. Results

### 3.1. Longitudinal Factor Analysis (LFA)

Model fit under strong factorial invariance was acceptable both when cognitive and functional limitation factors were estimated independently using unscaled scores and when they were estimated concurrently using standardized scaling. Model fit for the bivariate LFA was: Χ^2^(171) = 6090; TLI = .912; RMSEA = .053, 95%CI [.052, .054]. Standardized item-factor loadings for both cognitive ability and functional limitation were all strong (e.g., >.40) (Brown 2015). For cognitive ability, standardized loadings ranged from 0.445 (recall memory) to 0.850 (numeracy), confirming a strong emphasis on fluid processes. For functional limitation, standardized loadings ranged from 0.578 (IADL) to 0.820 (ADL). Results for the full bivariate LFA are reported in the Supplemental Materials.

Note that observed functional limitation items were positively skewed. We therefore estimated functional limitation factor scores (a) based on raw scaling, (b) based on standardized scaling, (c) following log-transformation, and (d) treating the items as categorical variables. Correlations between the functional limitation factor scores obtained by these different approaches were all very strong (*r* = .86–1.00). In the end, we standardized both the cognitive and functional limitation variables to support model convergence and consistency of interpretation. Treating the functional limitation variables as continuous (rather than categorical) also allowed for estimation using FIML, which accommodates missing data. Detailed information on these analyses are provided in the Supplemental Materials.

### 3.2. Univariate Trajectory Models

Fit indices for latent trajectory models are provided in Table 2. Out of the univariate trajectory analyses, spline models with knot points at 60/61 years, 64/65 years, and at 74/75 years did not converge for either process. Re-centering the data (from age 70 years to other knot points) and rescaling (from age in decades to age in years) had no effect on model convergence. For cognitive ability, the spline model with a knot point at age 70/71 years fit the data better than the intercept-only and linear models and showed acceptable overall fit: TLI = .971; RMSEA = .014, 95%CI [.014, .015]. For functional limitation, the spline model with a knot point at age 70/71 also fit the data best, with acceptable overall fit: TLI = .927; RMSEA = .021, 95%CI [.019, .022].

On average, univariate models showed that per-decade decline in cognitive ability (standardized scaling, centered at age 70 years) was significant prior to age 70 years (Est. = −.027, S.E. = .009, *p* = .002) followed by a steeper decline post-age 70 years (Est. = −.187, S.E. = .013, *p* < .001). On average, per-decade increase in functional limitation (standardized scaling, centered at age 70 years) prior to age 70 years was significant (Est. = .112, S.E. = .011, *p* < .001) followed by a steeper increase in limitations post-age 70 years (Est. =.452, S.E.= .022, *p* < .001). The estimated trajectories are shown in Figure 1.

Significant between-person variation was found for most latent trajectory parameters (i.e., intercepts and slopes). However, variability was much more pronounced for intercepts and post-age 70 slopes than for pre-age 70 slopes. For cognition, variances were as follows: Intercept (Est. = .521, S.E. = .017), pre-age 70 slope (Est. = .086, S.E. = .016), post-age 70 slope (Est. = .284, S.E. = .032). For functional limitation, variances were as follows: Intercept (Est. = .755, S.E. = .058), pre-age 70 slope (Est. = .096, S.E. = .045), post-age 70 slope (Est. = 1.147, S.E. = .119). Importantly, between person variation in the pre-age 70 slope for functional limitation was non-significant (*p* = .031).

### 3.3. Bivariate Trajectory Models

**Figure 2:**
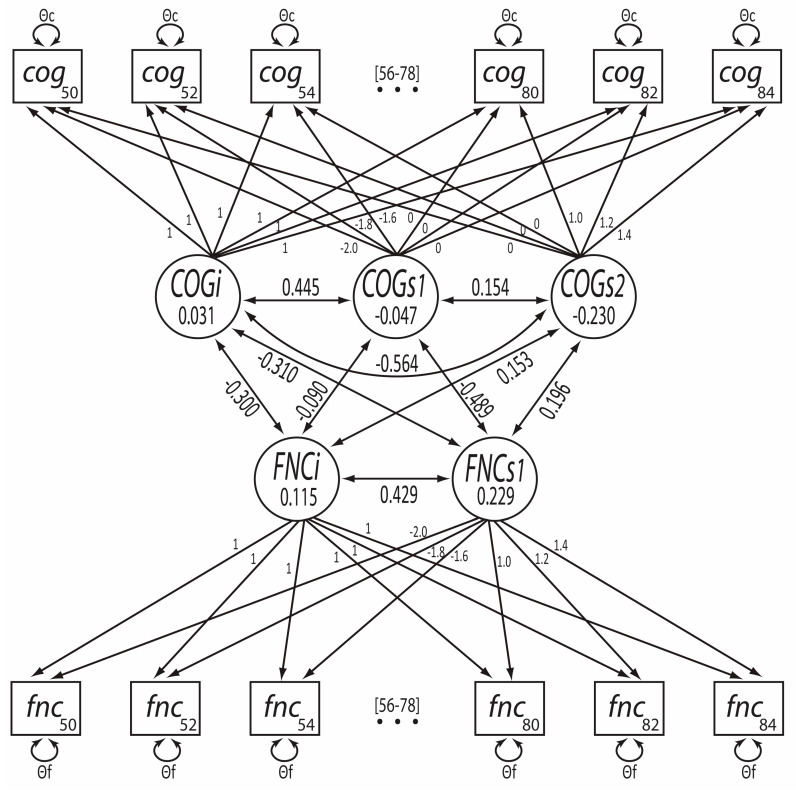
Path diagram of the best-fitting bivariate trajectory model. COG = Cognition Factor. FNC = Functional Limitation Factor. Subscripts for these factors (i, s1, s2) correspond to latent intercepts and slopes. Numerical values shown beneath factor labels are the estimated means. Numerical values on pathways connecting the factors (double-headed arrows) are correlations. Change in cognitive ability was modeled using a linear spline parameterization with two slopes pre- and post-knot-point at age 70 years. Change in functional limitation was modeled as a single linear slope (bivariate models with splines for functional limitations did not converge). Slopes’ loadings for both processes were scaled in decades, centered at age 70 years. Not shown due to space considerations: paths linked to observations at ages 56–78 years (as indicated by the placeholder ellipses, …), within-timepoint covariances between observed cognitive ability and functional limitation scores, and latent mean structure.

The bivariate trajectory model using splines for both cognitive and functional limitation did not converge; therefore, we tested combinations of linear slope/non-linear spline models (Table 2). Of these models, those with a non-linear spline for functional limitation all showed problems with model convergence. The best bivariate model was that with a non-linear spline for cognition and a linear slope for functional limitation. The path diagram for this model is shown in Figure 2. Parameter estimates are provided in Table 3 and are also shown in Figure 2.

Estimated per-decade declines in cognitive ability from the bivariate model were more pronounced than those from the univariate model but similarly showed that a slight but significant pre-age 70 decline (Est. = −.047, S.E. = .011, *p* < .001) was followed by a steeper decline post-age 70 years (Est. = −.230, S.E. = .016, *p* < .001). The per-decade linear increase in functional limitation was again significant (Est. = .229, S.E. = .008, *p* < .001). Between-person variation for all intercepts and slopes was significant in the bivariate model. Within-process correlations showed that higher baseline cognition (intercept) was positively correlated with change in cognition pre-age 70 years (*r* = .445) and negatively correlated with change in cognition post-age 70 years (*r* = −.564). In contrast, higher baseline functional limitation was positively correlated (*r* = .429), with a linear change in functional limitation across the observed age range.

Across processes, the intercept of cognitive ability was moderately negatively correlated with both the intercept (*r* = −.300, *p* < .001) and slope (*r* = −.310, *p* < .001) of functional limitation. The intercept of functional limitation was not significantly correlated with the pre-age 70 slope of cognitive ability (*r* = −.090, *p* = .079) but was weakly positively correlated with the post-age 70 slope of cognitive ability (*r* = .153, *p* < .001). The slope of functional limitation was moderately negatively correlated with the pre-age 70 slope of cognitive ability (*r* = −.489, *p* < .001) and weakly positively correlated with the post-age 70 slope of cognitive ability, although not significantly (*r* = .196, *p* = .022).

## 4. Discussion

We observed significant average declines in general fluid cognition and significant increases in functional limitation between ages 50–70 and from age 70–85 years. The average worsening in both processes was markedly steeper during the latter age window. Importantly, we also observed significant individual variation in cognitive and functional changes, and this was more pronounced post-age 70 years. Increasing functional limitation from age 50–85 years was significantly positively correlated with cognitive decline prior to age 70 years but not significantly correlated with cognitive decline post-age 70 years. Taken together, these outcomes point toward the importance of assessing changes both in cognition and functional limitations beginning in middle age, a period during which screening, prevention, and intervention strategies targeting either domain are likely to beneficially impact the other.

The observed average trajectory of general fluid cognition was consistent with previous evidence showing that individual differences in changes in fluid abilities are detectable earlier in the lifespan (i.e., before/during middle age) than changes in crystallized abilities (i.e., vocabulary), and that cognitive declines accelerate in later life (Baltes et al. 2006; Schaie 2005). Prior studies of functional limitation similarly provide evidence of age-related linear worsening, especially in motor skills and muscle strength (Hall et al. 2017; Liao and Chang 2020).

Within-process intercept/slope associations indicated that higher baseline cognitive ability was strongly positively correlated with the pre-age 70 cognitive slope and strongly negatively correlated with the post-age 70 cognitive slope. Individuals who start at a higher level of cognitive ability may better maintain that ability into later life, at which point they evince comparatively sharper decline (a type of selection effect). That is, individuals with higher baseline cognitive ability may be afforded lifestyle and health advantages that forestall decline (i.e., less negative slope pre-age 70 years), whereas in later life (post-age 70 years) pathological processes may override such advantages, giving the appearance of more rapid decline (i.e., because for such individuals there is “more to lose”). This cognitive trajectory pattern has been documented by several other studies and may be related to cognitive compensation strategies and/or protective factors associated with higher educational attainment (Baltes et al. 2006; Scarmeas et al. 2006). Baseline functional limitation was positively correlated with its own slope, indicative of a self-perpetuating state: if a person has more functional limitation at baseline, they tend to experience steeper increases in functional limitation as they age.

A salient bivariate outcome was the strong correlation between functional decline and midlife (but not later life) cognitive decline. This went against our initial hypothesis that these processes would be more strongly correlated in later life. Results from the univariate models showed that between-person variability in functional limitation slopes was only significant post-age 70 years, whereas significant variability in cognitive slopes was present both before and after age 70 years. It is not entirely surprising then that bivariate models failed to converge when including non-linear spline slopes for functional limitation. That said, we were able to obtain parameter estimates (without standard errors) from the model wherein both processes were modeled using non-linear splines. Slope-slope associations were all weak except for one: a strong negative correlation (*r* = −.529) between the pre-age 70 slope for cognition and post-age 70 slope for functional limitation. In other words, better retention of cognitive ability prior to age 70 years was linked to less worsening in functional limitation following age 70 years. We must of course take this interpretation with a large grain of salt given that it hinges on evidence from a non-convergent model.

Notwithstanding, comparative bivariate results, on the whole, suggest (a) that in midlife, variability in change in cognition may be more reliably detected than variability in change in functional limitation, (b) that cognitive decline during midlife may be indicative of elevated risk for of increasing functional limitation, and (c) that etiological pathways affecting cognitive decline in midlife vs. later life may differ. On this latter point, cognitive performance during midlife may be especially sensitive to system-wide factors, such as metabolic and/or cerebrovascular diseases, that are also implicated in functional limitation. In contrast, cognitive performance in later life may be more strongly affected by proximal factors (e.g., amyloid plaques; Gottesman et al. 2017) that are less closely linked to functional limitation per se. Additional research will be needed to clarify the underlying neurobiological processes and related risk/protective influences differentially affecting cognitive and functional processes across middle and later adulthood. Studies which leverage dynamic models of temporal dependencies between cognitive and functional processes may be especially helpful for this purpose.

The strengths of this study are its use of a large, nationally representative sample with a substantial percentage of middle-aged adults (50–65 years of age), a demographic often lacking in similar studies. Modeling both cognitive ability and functional limitation as factors is a strength. To our knowledge, we are the first to estimate age-related changes in the fluid cognitive measures introduced in the HRS between 2010–2016—and further to examine these changes in relation to changes in functional limitation, similarly modeled as a latent variable. We have made available for download the estimated factor scores for each process (general fluid cognition and functional limitation at HRS waves 2010, 2012, and 2016) contingent on HRS approval of data use. Limitations in this study are that some data were sparse at certain age ranges (i.e., 80–85 years), and function limitation variables were collected only via self-report (and were positively skewed). The cognitive tests in our analyses include measures of fluid abilities, which were not collected prior to year 2010 in the HRS. These tests are an improvement from prior waves; however, they are brief in nature and were intended to be collected over the phone or face-to-face in large epidemiologic studies (Fisher et al. 2014). Additionally, we did not adjust for cognitive re-test effects, nor did we examine further explanatory variables. Our goal was first and foremost to model the bivariate fluid cognition-functional limitation trajectories as a function of chronological age and to facilitate a closer examination of these processes (and variables) in future HRS studies.

## 5. Conclusions

Findings from this study demonstrate age-related declines in both general fluid cognition and functional ability—and differential patterns of individual variation in cognitive and functional worsening, from middle age into later life (50–85 years)—in participants from the Health and Retirement Study (Waves 2010, 2012, and 2016). Results further highlight the importance of attending to changes in cognition during midlife, when they appear to be most strongly predictive of later worsening in functional ability, and when early screening and intervention measures may help to promote autonomy and quality of life in later adulthood.

## Figures and Tables

**Table 1:** Sample descriptive statistics (N = 14,489) including demographic, cognitive, and functional limitation values.

Variable	Summary Statistics					
	All Comers (N = 14,489)			Completers (N = 11,033)		
Age in years as of 2010	*M* = 64.9, *SD* = 9.6			*M* = 64.3, *SD* = 9.2		
Women	*n* = 8126 (56.1%)			*n* = 6372 (57.8%)		
Years of education	*M* = 13.1, *SD* = 2.9			*M* = 13.2, *SD* = 2.9		
	**Mean (SD) by Wave**					
	**2010**	**2012**	**2016**	**2010**	**2012**	**2016**
Cognitive Ability	*n* = 14,489	*n* = 13,277	*n* = 10,900			
Fluid Reasoning	497.3 (43.4)	522.8 (31.4)	521.8 (31.3)	499.2 (142.8)	523.9 (31.0)	521.9 (31.3)
Executive Function	3.1 (1.9)	3.1 (1.9)	3.1 (1.9)	3.1 (1.9)	3.1 (1.9)	3.1 (1.9)
Category fluency	17.4 (7.1)	17.9 (7.3)	16.7 (6.6)	17.8 (7.1)	18.2 (7.4)	16.7 (6.6)
Recall Memory	10.1 (3.3)	10.0 (3.4)	9.7 (3.5)	10.3 (3.2)	10.1 (3.3)	9.8 (3.5)
Factor Score	−0.1 (0.9)	0.1 (0.8)	0.1 (0.7)	−0.1 (0.8)	0.1 (0.8)	0.1 (0.8)
Functional Limitation	*n* = 14,489	*n* = 13,430	*n* = 11,205			
ADL	0.3 (0.9)	0.4 (1.0)	0.5 (1.2)	0.3 (0.8)	0.3 (0.8)	0.5 (1.2)
IADL	0.1 (0.4)	0.1 (0.5)	0.2 (0.7)	0.1 (0.4)	0.1 (0.5)	0.2 (0.7)
Mobility	2.6 (2.7)	2.7 (2.7)	3.0 (2.9)	2.5 (2.6)	2.6 (2.6)	3.0 (2.9)
Factor Score	−0.1 (0.9)	−0.1 (1.0)	0.1 (1.1)	−0.2 (0.8)	−0.2 (0.9)	0.1 (1.2)

Note: All comers = individuals present at one or more waves. Completers = individuals who were present at all waves. Reported n’s by wave for all comers = participants who completed one or more measures within the given task category (cognitive ability, functional limitation). Means and standard deviations (SDs) for variables other than factor scores are provided in raw (observed, unscaled) metrics. ADL = activities of daily living; IADL = instrumental activities of daily living; Mobility = composite of 11 mobility related questions. Factor scores were estimated using scaled variables, as described in Methods.

**Table 2:** Model fit statistics for the latent trajectory models.

Model	Χ^2^(*df*)	TLI	RMSEA [95%CI]	AIC
Cognitive Ability				
Intercept-only	1124 (186)	.948	.019 [.018, .020]	80,554
Linear Slope	906 (183)	.959	.017 [.016, .018]	80,343
Spline, KP = 70/71y	670 (179)	.971	.014 [.014, .015]	80,114
Functional limitation				
Intercept-only	3731 (186)	.781	.036 [.035, .037]	96,756
Linear Slope	2120 (183)	.880	.027 [.025, .028]	95,151
Spline, KP = 70/71y	1346 (179)	.927	.021 [.019, .022]	94,386
Bivariate				
Cognitive Ability (linear slope) + Functional limitation (linear slope)	3265 (685)	.915	.017 [.016–.017]	200,094
Cognitive Ability (spline, KP = 70/71) + Functional limitation (linear slope)	3005 (679)	.922	.016 [.015–.016]	199,846

Note: KP = knot point. Out of the spline trajectory models, only those with a knot point at 70/71 years converged. For space considerations, the non-converging spline models are not listed. TLI = Tucker–Lewis index; RMSEA = root mean square error of approximation; AIC = Akaike information criterion.

**Table 3:** Parameter estimates for the bivariate trajectories of cognitive ability and functional limitation.

Parameter	Estimate	S.E.	Z	*p*
Means				
Intercept of Cognitive Ability	.031	.012	2.673	.008
Slope (pre-70y) of Cognitive Ability	−.047	.011	−4.442	<.001
Slope (post-70y) of Cognitive Ability	−.230	.016	−14.376	<.001
Intercept of Functional limitation	.115	.009	12.293	<.001
Slope of Functional limitation	.229	.008	27.195	<.001
Variances				
Intercept of Cognitive Ability	.777	.021	36.731	<.001
Slope (pre-70y) of Cognitive Ability	.142	.021	6.716	<.001
Slope (post-70y) of Cognitive Ability	.413	.035	11.861	<.001
Intercept of Functional limitation	.769	.016	49.143	<.001
Slope of Functional limitation	.150	.013	11.782	<.001
Residual of Cognitive Ability	.313	.003	94.410	<.001
Residual of Functional limitation	.475	.005	101.098	<.001
	**Std. Estimate**		**Z**	***p***
Correlations, Within Processes				
Intercept of Cognitive Ability ~ Slope (pre-70y) of Cognitive Ability	.445		7.158	<.001
Slope (post-70y) of Cognitive Ability	−.564		−12.841	<.001
Slope (pre-70y) of Cognitive Ability ~ Slope (post-70y) of Cognitive Ability	.154		0.270	.787
Intercept of Functional limitation ~ Slope of Functional limitation	.429		17.132	<.001
Correlations, Across Processes				
Intercept of Cognitive Ability ~ Intercept of Functional limitation	−.300		−18.420	<.001
Slope of Functional limitation	−.310		−8.165	<.001
Intercept of Functional limitation ~ Slope (pre-70y) of Cognitive Ability	−.090		−1.759	.079
Slope (post-70y) of Cognitive Ability	.153		3.535	<.001
Slope of Functional limitation ~ Slope (pre-70y) of Cognitive Ability	−.489		−5.353	<.001
Slope (post-70y) of Cognitive Ability	.196		2.289	.022
Residual of Cognitive Ability ~ Residual of Functional limitation	.007		2.238	.025

Note: Slopes were scaled as change per decade. Slopes for Cognitive Ability were estimated pre- and post-knot point (50–70 years, and 70–85 years). Slope for Functional limitation spanned the observed age range of 50–85 years and was centered at age 70 years. ~ designates a correlation. S.E. = Standard Error, Std. Estimate = Standardized Estimate.

## Data Availability

Data for the Health and Retirement Study are publicly available at https://hrs.isr.umich.edu.

## Supplementary Materials

The following supporting information can be downloaded at: https://www.mdpi.com/article/10.3390/jintelligence11040065/s1, Figure S1.1. Histograms of participants’ means (across measurement waves) of cognitive scores; Figure S1.2. Histograms of participants’ means (across measurement waves) of functional limitation scores; Table S2.1. Correlations between cognitive items across waves; Table S2.2. Correlations between functional limitation items across waves; Table S3.1. Fit statistics for the longitudinal factor models; Table S3.2. Parameter estimates for the best-fitting bivariate longitudinal factor model.
