# Supplementary material for: Age-Related Trajectories of General Fluid Cognition and Functional Decline in the Health and Retirement Study: A Bivariate Latent Growth Analysis
Source: J Intell. 2023 Mar 29;11(4):65. doi: 10.3390/jintelligence11040065 (PMC10144147; doi:10.3390/jintelligence11040065)
Supplement: Supplementary file 1 [file jintelligence-11-00065-s001.zip › jintelligence-2237422-supplementary.pdf]

Supplemental Materials (SM)

S1 Histograms for observed longitudinal variables’ means and corresponding factor scores

We calculated each participant’s mean score across waves for each of the observed cognitive measures and for the corresponding factor score. The histograms below show these scores sorted by age quantiles (based on observed participant ages: < 56 years, 56-64 years, 64-73 years, and > 73 years of age). Older individuals, on average, showed lower levels of cognitive ability and higher levels of functional limitations across measurement waves. There was evidence of a ceiling effect for cognitive executive functioning. Functional limitation variables all showed positive skew.

Figure S1.1. Histograms of participants’ means (across measurement waves) of cognitive scores.

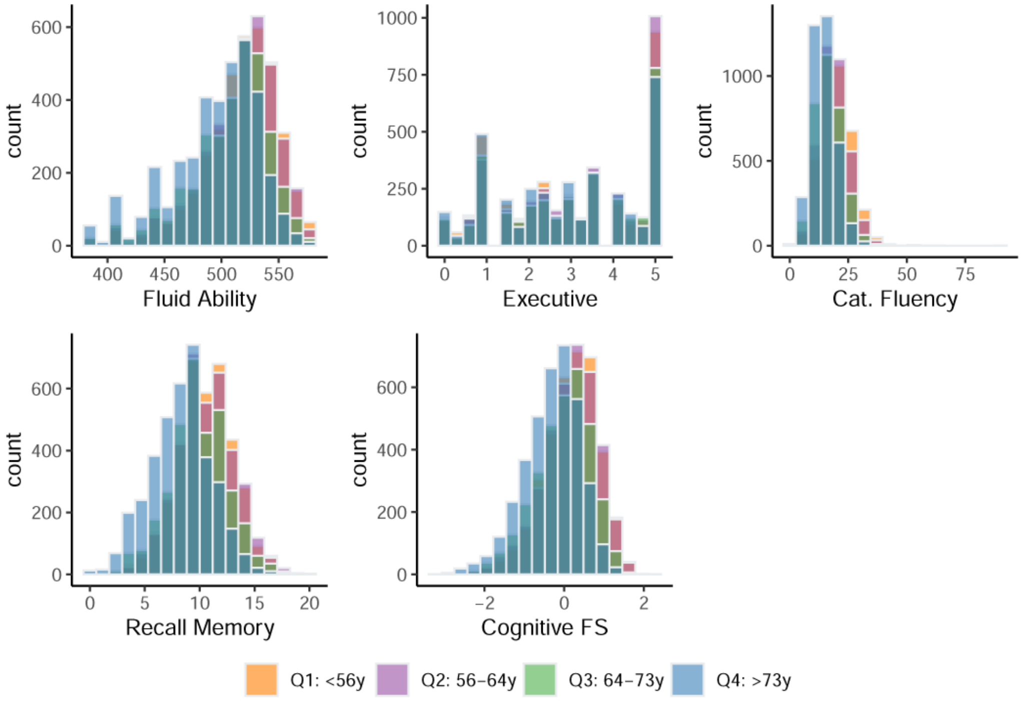

Figure S1.2. Histograms of participants’ means (across measurement waves) of functional limitation scores.

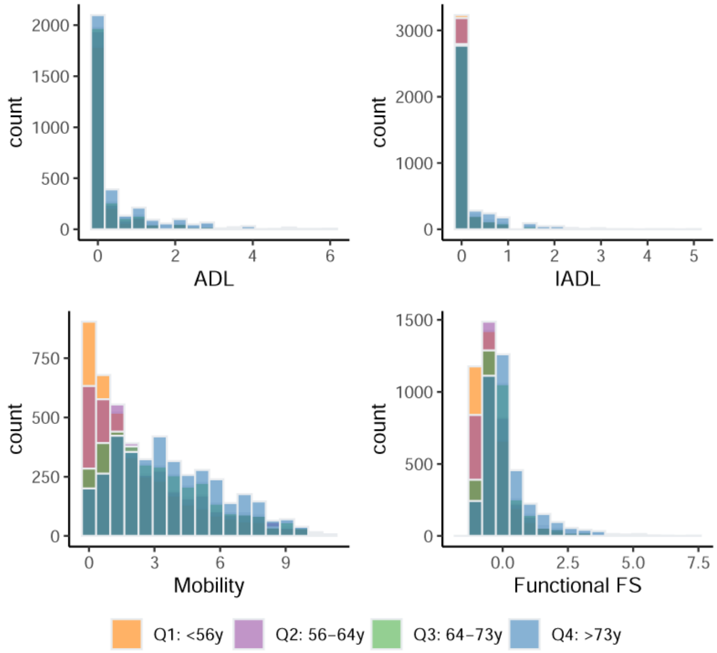

S2 Longitudinal correlations between observed factor indicators

Table S2.1 Correlations between cognitive items across waves

	Numr. 0	Numr. 2	Numr. 6	Execu. 0	Execu. 2	Execu. 6	Flncy. 0	Flncy. 2	Flncy. 6	Mmry. 0	Mmry. 2	Mmry. 6
Numr.0		0.66	0.68	0.39	0.40	0.41	0.39	0.38	0.36	0.36	0.34	0.35
Numr.2	0.58		0.67	0.39	0.40	0.42	0.36	0.39	0.37	0.33	0.34	0.34
Numr.6	0.62	0.62		0.39	0.39	0.43	0.37	0.39	0.40	0.34	0.33	0.38
Execu.0	0.31	0.32	0.33		0.48	0.46	0.22	0.23	0.22	0.24	0.21	0.21
Execu.2	0.28	0.32	0.33	0.44		0.47	0.21	0.23	0.22	0.22	0.23	0.23
Execu.6	0.32	0.35	0.39	0.40	0.41		0.24	0.24	0.25	0.25	0.24	0.27
Flncy.0	0.30	0.28	0.33	0.18	0.11	0.20		0.61	0.57	0.34	0.32	0.33
Flncy.2	0.29	0.32	0.36	0.19	0.13	0.18	0.56		0.61	0.34	0.36	0.35
Flncy.6	0.29	0.29	0.34	0.16	0.12	0.21	0.56	0.59		0.33	0.34	0.39
Mmry. 0	0.25	0.27	0.26	0.17	0.15	0.18	0.28	0.26	0.27		0.49	0.47
Mmry. 2	0.25	0.26	0.29	0.14	0.16	0.19	0.27	0.27	0.30	0.41		0.54
Mmry. 6	0.23	0.26	0.32	0.16	0.16	0.20	0.28	0.31	0.32	0.43	0.51	

Note: Numr = Numeracy. Execu = executive function. Flncy = category fluency. Mmry = recall memory. Numeric suffixes show measurement wave year: .0 = 2010, .2 = 2012, and .6 = 2016. Correlations estimated case-wise are shown in the lower triangular section. Correlations estimated pairwise are shown in the upper triangular matrix.

Table S2.2 Correlations between functional limitation items across waves

	ADL.0	ADL.2	ADL.6	IADL.0	IADL.2	IADL.6	Mblty.0	Mblty.2	Mblty.6
ADL.0		0.50	0.37	0.39	0.32	0.24	0.48	0.38	0.31
ADL.2	0.45		0.43	0.30	0.43	0.29	0.38	0.49	0.36
ADL.6	0.36	0.42		0.23	0.33	0.48	0.30	0.33	0.52
IADL.0	0.30	0.23	0.22		0.43	0.30	0.30	0.26	0.22
IADL.2	0.26	0.33	0.27	0.43		0.40	0.28	0.32	0.26
IADL.6	0.22	0.23	0.40	0.28	0.38		0.26	0.28	0.37
Mblty.0	0.41	0.32	0.28	0.27	0.24	0.26		0.74	0.66
Mblty.2	0.29	0.41	0.30	0.23	0.29	0.28	0.62		0.70
Mblty.6	0.24	0.27	0.45	0.20	0.21	0.35	0.50	0.56	

Note: ADL = difficulty in activities of daily living. IADL = difficulty in instrumental activities of daily living. Mblty = difficulties with mobility. Numeric suffixes show measurement wave year: .0 = 2010, .2 = 2012, and .6 = 2016. Correlations estimated case-wise are shown in the lower triangular section. Correlations estimated pairwise are shown in the upper triangular matrix.

S3 Longitudinal Structural Factor Analyses

We used longitudinal factor analysis (LFA) to summarize individuals' cognitive ability and functional limitation as factor scores at each HRS measurement wave (2010, 2012, 2016). Cognition was assessed by numeracy, executive function, category fluency, and recall memory. Functional limitation was indicated by difficulty performing activities of daily living (ADL), difficulty performing instrumental activities of daily living (IADL), and mobility difficulties. First, we ran univariate analyses for cognitive and functional limitation variables. These models were applied to both sets of variables (a) scaled in their original raw metrics and (b) after standardization based on the means and standard deviations at the first (2010) measurement wave. To address positive skew in the functional limitation variables, we ran additional models (c) following log-transformation of the functional limitation items, and (d) treating the functional limitation items as ordered categorical variables. We then compared the model fits and the correlations of the extracted factor scores across these analyses. We ran additional bivariate models (e) with both sets of variables in standardized scaling, and (f) with cognitive variables in raw scaling and functional limitation variables specified as ordered/categorical.

For continuous outcomes, strong factorial invariance was imposed across measurement occasions to ensure consistency of factor-item representation and scaling (Widaman et al., 2010). For categorical items, equivalent constraints were placed on item thresholds, loadings, and intercepts under theta parameterization (Wu & Estabrook, 2016). Models applied to continuous data only were fit with full information maximum likelihood (FIML) with Huber-White robust estimation (Huber, 1967) to handle missing values and non-normality. Models fit to data with categorical items were estimated using weighted least squares with mean- and variance-adjusted test statistics (WLSMV). Because WLSMV does not accommodate missingness, we imputed a single complete data set for these latter analyses. The imputation included the cognitive and functional limitation variables, as well as chronological age and biological sex variables.

Model fit statistics are provided in Table S3.1 below. Absolute criteria (CFI, TLI, RMSEA) generally indicated acceptable fit (Hu & Bentler, 1999; Steiger, 1990). Standardized factor loadings were in all cases strong (>.40; Brown, 2015). Cognition factor scores based on items in raw scaling were perfectly correlated with those based on standardized items (r=1.00). Similarly, functional limitation factors estimated from raw, scaled, and log-transformed data were nearly perfectly correlated (r=.97). Functional limitation factor scores based on ordinal scaling were correlated very strongly with those based on raw and standardized scaling (r=.86). In other words, factor score estimates were highly consistent across different item scalings. Of the bivariate models, that based on standardized items fit the data very well and yielded factor scores, whereas the software (R Core Team, 2021; Rosseel, 2012) "hung" during factor score estimation for the model applied to mixed data types, despite evidence of even better overall fit of the latter.

In the end, we chose to extract factor scores from the bivariate model applied to standardized cognitive and functional limitation items. Because the data were longitudinal and showed clear participant attrition across waves, we prioritized accounting for missingness, and the use of FIML was preferable to WLSMV for this purpose. Concurrent estimation of scores for both processes also allowed for cognitive scores to inform missingness in functional limitation scores, and vice versa—and was also consistent with bivariate growth models in subsequent analyses. The very high correlations of factor scores estimated under different variable scalings and the large sample size were further reassurance that the decision to work with fully standardized variables was not unreasonable in this instance. Parameter estimates for the corresponding bivariate model are shown below in Table S3.2.

Table S3.1 Fit statistics for the longitudinal factor models

Model	X^2 (df)	CFI	TLI	RMSEA [95%CI]	AIC
Univariate: Cognition (COG)					
Items in raw scale	5,031 (50)	.898	.866	.084 [.082, .086]	910,831
Items z-scaled	5,018 (50)	.900	.867	.083 [.081, .085]	359,883
Univariate: Functional Limitation (FNC)					
Items in raw scale	627 (22)	.973	.955	.062 [.058, .066]	273,311
Items z-scaled	627 (22)	.973	.955	.062 [.058, .066]	270,042
Items log-transformed	670 (22)	.978	.964	.053 [.050, .056]	87,106
Ordinal items [imputed]	626 (41)	.978	.955	.031 [.029, .034]	
Bivariate: COG & FNC					
COG & FNC items all z-scaled	6,090 (171)	.928	.912	.053 [.052, .054]	628,614
COG (z-scaled), FNC (ordinal) [imputed]	4,612 (91)	.957	.970	.059 [.057, .060]	

Table S3.2 Parameter estimates for the best-fitting bivariate longitudinal factor model

Parameter	Estimate	S.E.	Std. Est	*p*
**Factor Loadings**				
Cognitive Ability ~				
Numeracy	1.000		[0.672, 0.850]	< .001
Executive Function	0.729	0.014	[0.492, 0.504]	< .001
Category Fluency	0.698	0.020	[0.477, 0.502]	< .001
Recall Memory	0.701	0.021	[0.445, 0.478]	< .001
Functional Limitation ~				
ADL difficulties	1.000		[0.787, 0.820]	< .001
IADL difficulties	0.839	0.022	[0.578, 0.621]	< .001
Mobility limitations	0.730	0.020	[0.594, 0.715]	< .001
**Intercepts**				
Observed Variables				
Numeracy	0.345	0.004	[0.339, 0.437]	< .001
Executive Function	-0.042	0.005	[-0.043, -0.042]	< .001
Category Fluency	0.005	0.006	[0.005, 0.005]	0.422
Recall Memory	-0.056	0.006	[-0.056, -0.053]	< .001
ADL difficulties	-0.163	0.004	[-0.166, -0.121]	< .001
IADL difficulties	0.123	0.006	[0.078, 0.118]	< .001
Mobility limitations	0.099	0.006	[0.089, 0.104]	< .001
Factors				< .001
Cognitive Ability (2010)	-0.118	0.008	-0.172	< .001
Cognitive Ability (2012)	0.116	0.007	0.180	< .001
Cognitive Ability (2016)	0.044	0.007	0.065	< .001
Functional Limitation (2010)	-0.127	0.005	-0.164	< .001
Functional Limitation (2012)	-0.064	0.006	-0.071	< .001
Functional Limitation (2016)	0.156	0.010	0.142	< .001
**Variances**				
Factors				
Cognitive Ability (2010)	0.468	0.013	1.000	< .001
Cognitive Ability (2012)	0.417	0.010	1.000	< .001
Cognitive Ability (2016)	0.466	0.010	1.000	< .001
Functional Limitation (2010)	0.600	0.030	1.000	< .001
Functional Limitation (2012)	0.794	0.037	1.000	< .001
Functional Limitation (2016)	1.194	0.054	1.000	< .001
Residuals/Uniquenesses				
Numeracy	[0.179, 0.567]	[0.009, 0.016]	[0.277, 0.548]	< .001
Executive Function	[0.729, 0.777]	[0.007, 0.008]	[0.746, 0.775]	< .001
Category Fluency	[0.673, 0.803]	[0.016, 0.027]	[0.748, 0.798]	< .001
Recall Memory	[0.778, 0.875]	[0.012, 0.016]	0.772, 0.802]	< .001
ADL difficulties	[0.368, 0.618]	[0.019, 0.033]	[0.341, 0.380]	< .001
IADL difficulties	[0.673, 1.677]	[0.036, 0.077]	[0.614, 0.666]	< .001
Mobility limitations	[0.588, 0.609]	[0.014, 0.016]	[0.488, 0.648]	< .001

Table S3.2 continues next page...

Table S3.2 (Cont.'d)

Parameter	Estimate	S.E.	Std. Est	*p*
**Factor Covariances**				
Cognitive Ability (2010) ~~				
Cognitive Ability (2012)	0.557	0.027	0.808	< .001
Cognitive Ability (2016)	0.567	0.027	0.670	< .001
Functional Limitation (2010)	-0.164	0.008	-0.310	< .001
Functional Limitation (2012)	-0.201	0.009	-0.331	< .001
Functional Limitation (2016)	-0.280	0.012	-0.374	< .001
Cognitive Ability (2012) ~~				
Cognitive Ability (2016)	0.733	0.034	0.753	< .001
Functional Limitation (2010)	-0.156	0.008	-0.313	< .001
Functional Limitation (2012)	-0.197	0.009	-0.342	< .001
Functional Limitation (2016)	-0.266	0.012	-0.377	< .001
Cognitive Ability (2016) ~~				
Functional Limitation (2010)	-0.167	0.009	-0.316	< .001
Functional Limitation (2012)	-0.208	0.010	-0.342	< .001
Functional Limitation (2016)	-0.309	0.014	-0.414	< .001
Functional Limitation (2010) ~~				
Functional Limitation (2012)	0.557	0.027	0.808	< .001
Functional Limitation (2016)	0.567	0.027	0.670	< .001
Functional Limitation (2012) ~~				
Functional Limitation (2016)	0.733	0.034	0.753	< .001

Note: For space-saving purposes, square brackets denote the range, [min, max], of estimates across measurement occasions when these estimates are not provided as individual rows. Thus, for the factor loadings, the bracketed values show the ranges of standardized factor loadings across measurement waves.

References:

Brown, T. A. (2015). *Confirmatory factor analysis for applied research* (2nd Edition ed.). New York: Guilford Press.

Hu, L. t., & Bentler, P. M. (1999). Cutoff criteria for fit indexes in covariance structure analysis: Conventional criteria versus new alternatives. *Structural Equation Modeling: A Multidisciplinary Journal, 6*(1), 1-55. doi:10.1080/10705519909540118

R Core Team (2021). *A language and environment for statistical computing*. https://www.R-project.org/

Rosseel, Y. (2012). Lavaan: An R Package for Structural Equation Modeling. *Journal of Statistical Software, 48*(2), 1 - 36. doi:10.18637/jss.v048.i02

Steiger, J. H. (1990). Structural Model Evaluation and Modification: An Interval Estimation Approach. *Multivariate Behav Res, 25*(2), 173-180. doi:10.1207/s15327906mbr2502_4

Widaman, K. F., Ferrer, E., & Conger, R. D. (2010). Factorial Invariance within Longitudinal Structural Equation Models: Measuring the Same Construct across Time. *Child Dev Perspect*, *4*(1), 10-18. doi:10.1111/j.1750-8606.2009.00110.x

Wu, H., & Estabrook, R. (2016). Identification of confirmatory factor analysis models of different levels of invariance for ordered categorical outcomes. *Psychometrika*, *81*(4):1014-1045. doi: 10.1007/s11336-016-9506-0
